# Binding Mechanism of Inhibitors to Heat Shock Protein 90 Investigated by Multiple Independent Molecular Dynamics Simulations and Prediction of Binding Free Energy

**DOI:** 10.3390/molecules28124792

**Published:** 2023-06-15

**Authors:** Fen Yang, Yiwen Wang, Dongliang Yan, Zhongtao Liu, Benzheng Wei, Jianzhong Chen, Weikai He

**Affiliations:** 1 School of Information Science and Electrical Engineering, Shandong Jiaotong University, Jinan 250357, China; fen_yang0223@163.com (F.Y.);; 2 School of Aeronautics, Shandong Jiaotong University, Jinan 250357, China; 3 School of Science, Shandong Jiaotong University, Jinan 250357, China; 4 Center for Medical Artificial Intelligence, Shandong University of Traditional Chinese Medicine, Qingdao 266112, China

**Keywords:** HSP90, multiple all-atom molecular dynamics simulations, hot interaction spots, MM-GBSA, principal component analysis

## Abstract

The heat shock protein (HSP90) has been an import target of drug design in the treatment of human disease. An exploration of the conformational changes in HSP90 can provide useful information for the development of efficient inhibitors targeting HSP90. In this work, multiple independent all-atom molecular dynamics (AAMD) simulations followed by calculations of the molecular mechanics generalized Born surface area (MM-GBSA) were performed to explore the binding mechanism of three inhibitors (W8Y, W8V, and W8S) to HSP90. The dynamics analyses verified that the presence of inhibitors impacts the structural flexibility, correlated movements, and dynamics behavior of HSP90. The results of the MM-GBSA calculations suggest that the selection of GB models and empirical parameters has important influences on the predicted results and verify that van der Waals interactions are the main forces that determine inhibitor–HSP90 binding. The contributions of separate residues to the inhibitor–HSP90 binding process indicate that hydrogen-bonding interactions (HBIs) and hydrophobic interactions play important roles in HSP90–inhibitor identifications. Moreover, residues L34, N37, D40, A41, D79, I82, G83, M84, F124, and T171 are recognized as hot spots of inhibitor–HSP90 binding and provide significant target sites of for the design of drugs related to HSP90. This study aims to contribute to the development of efficient inhibitors that target HSP90 by providing an energy-based and theoretical foundation.

## 1. Introduction

The heat shock protein (HSP) has been found to be highly conserved in biological evolution and widely exists in prokaryotes and eukaryotes [1]. Derived from a family member of HSP, HSP90 is an ATP-related molecular chaperone that plays a critical role in the maintenance of the conformation, stability, and function of extensive signaling proteins that function as pathways of cell proliferation, cell cycle progression, angiogenesis, invasion, and metastasis [2,3,4]. The family members of HSP have been known to be involved in the onset of neurodegenerative disorders and viral infections [5,6]. The inhibition of the HSP function has been found to induce proteasomal degradation of the oncogenic client proteins, thereby exerting control over the growth of cancer cells [6,7,8,9]. Currently, HSP90 is regarded as an important target of drugs designed towards the treatment of cancers. Moreover some HSP90 inhibitors have been developed and show antitumor, antiparasitic, and antiviral activity [9,10,11,12]. Therefore, insights into the interaction mechanism of inhibitors with HSP90 are important for the development of efficient inhibitors that target HSP90.

Based on previous reports, small-molecule inhibitors of HSP90 can have simultaneous influences on various abnormal signaling pathways relating to tumor cells, which possibly explains several issues related to cancer resistance [13,14,15,16]. According to animal model tests, the inhibition of the HSP90 function restores sensitivity to drug-resistant fungal pathogens, delays the evolution of drug resistance, and results in a clearance of otherwise lethal infections [17,18,19,20,21]. Despite the high efficiency of small-molecule inhibitors toward HSP90, the currently existing inhibitors of HSP90 generate common side effects, which prevents some novel inhibitors from application in clinical studies [22,23,24,25]. In fact, both inhibitor binding and HSP90 chaperone cycles lead to large conformational changes in the flexible structure of HSP90 [26,27,28], which makes the design of a drug that can relieve side effects challenging. Moreover, multiple reports have indicated that the conformational changes in the N-terminal ATP site of HSP90 play a key role in the inhibition of ATP hydrolysis and client processing [29,30,31,32]. It is well known that the thermodynamics and kinetics of conformational changes are essential for gaining insights into the molecular mechanism of inhibitor–HSP90 binding and for the development of clinically available drugs against cancers [33]. Therefore, a clarification of the conformational changes of HSP90 caused by inhibitor binding and the binding mechanism of inhibitors to HSP90 at atomic levels is of high importance for future drug design targeting HSP90.

The all-atom molecular dynamics (AAMD) simulation [34,35,36,37,38,39,40,41,42] is one of several emerging crossover biology tools; moreover, MD simulations have been employed to study conformational changes in biological macromolecules caused by inhibitor binding. The combination of MD simulations with calculations of binding free energy [43,44,45,46,47,48,49,50] has been adopted to explore the binding modes of inhibitors to targets. More interestingly, these methods have been employed to successfully decipher atomic-level molecular mechanisms with respect to the conformational alterations of HSP90 caused by the presence of inhibitors [51,52,53]. For example, Nazar et al. applied molecular docking and MD simulations to investigate an inhibition mechanism against HSP90, and their results suggested that residues near interacting active sites drive the binding and stability of the inhibitors [54]. An investigation of the effect of MD simulations on inhibitor–HSP90 complexes by Rezvani et al. revealed that hydrogen-bonding interactions (HBIs), and the hydrophobic and salt bridge interactions of inhibitors with HSP90, were determinant forces that exist in a complex formation [55]. Chen et al. used MD simulations and principal component analysis (PCA) to study the effect of structural difference in inhibitors of the conformational dynamics of HSP90, and their results indicated that inhibitor bindings exert significant effects on the conformational changes, internal dynamics, and motion modes of HSP90 [56]. Despite these successes, conformations sampled using conventional MD simulations are possibly trapped at a local minimization space, which results in an insufficient conformational sampling. Recently, multiple independent MD simulations [57,58,59,60,61] have been applied by different researchers to obtain rational conformation sampling, which has been verified by the work of Suruzhon et al. [62]. Thus, in the current study, multiple independent MD simulations are adopted to enhance the conformational sampling of HSP90.

**Figure 1:**
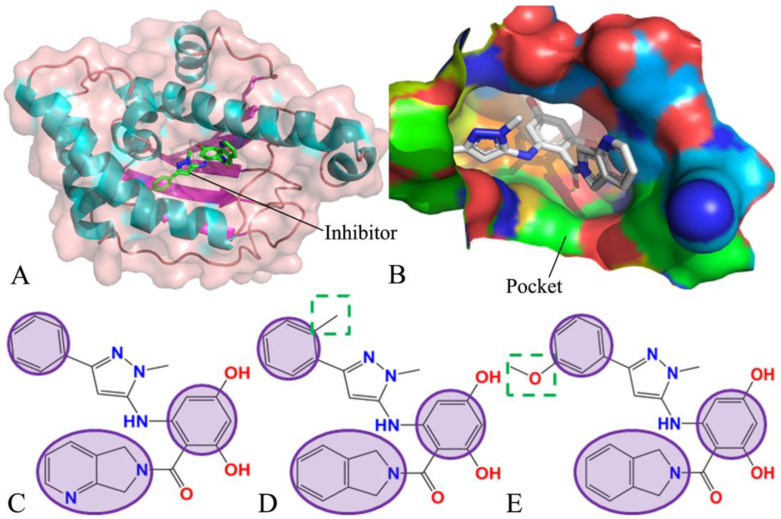
Molecular structures: (**A**) the inhibitor–HSP90 complex, in which HSP90 is represented using cartoon and surface modes and the inhibitor is displayed using stick modes; (**B**) binding pocket of inhibitor to HSP90, in which the pocket is depicted in surface styles, and (**C**–**E**) respectively correspond to the structures of W8Y, W8V, and W8S.

To reveal the molecular mechanism with respect to inhibitor–HSP90 binding, the *apo* HSP90 (without inhibitor binding) and three inhibitor-bound HSP90s, including inhibitors W8Y, W8V, and W8S [11], were selected for this current study. The structure of the inhibitor-bound HSP90 and the binding pocket of the HSP90 are respectively depicted in Figure 1A,B. The three inhibitors (W8Y, W8V, and W8S) share a common molecular structure scaffold and only exhibit small differences in structure (Figure 1C–E); however, they have obvious differences in their binding ability to HSP90. Specifically, the binding strengths of W8V and W8S to HSP90, when quantified by their respective EC50 values, were 65 nM and 281 nM [11]. A clarification of the molecular mechanism concerning the effect of small differences in structure on the binding ability of inhibitors is of significance in the successful development of efficient HSP90 inhibitors. To achieve this, multiple independent MD simulations, principal component analysis (PCA) [63,64,65,66], and binding free energy calculations were coupled to probe for conformational changes in HSP90 caused by inhibitor binding and to examine the binding ability of inhibitors to HSP90. This work is expected to provide useful theoretical guidance and serve as an energy-based foundation for the design of drugs targeting HSP90.

## 2. Results and Discussion

### 2.1. Stability of Molecular Dynamics Simulations

**Figure 2:**
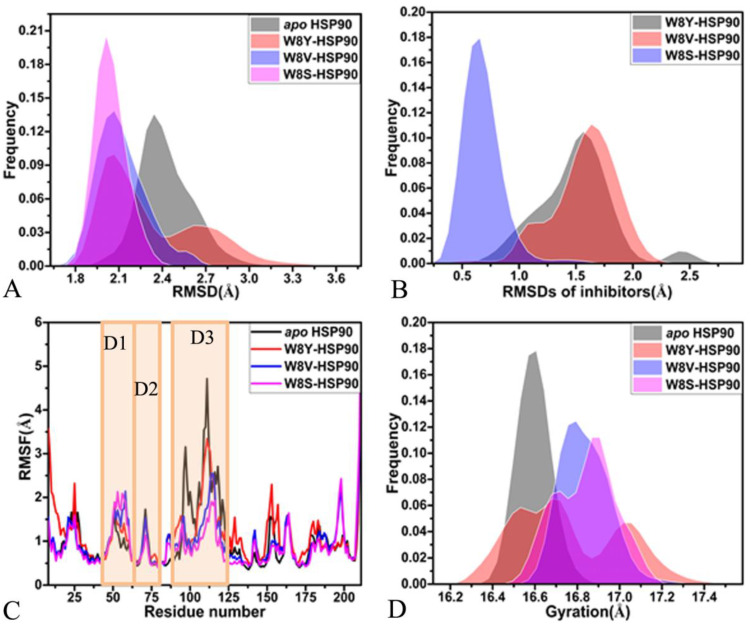
Structural fluctuations: (**A**) frequency distribution of the HSP90 RMSDs after the equilibrium of the systems; (**B**) frequency distribution of the inhibitor RMSDs after the equilibrium of the systems; (**C**) RMSFs of HSP90 calculated using the coordinates of the Cα atoms and (**D**) the frequency distribution of the gyration for HSP90.

To assess the structural stability of four systems during three independent MD simulations, root-mean-square deviations (RMSDs) of backbone atoms from HSP90 were calculated and compared with the initial optimized structures, the results of which are depicted in the supporting information (Figure S1). It was observed that the four simulated systems reached their equilibrium after 300 ns of the MD simulations. To facilitate the postprocessing analyses, the equilibrated sections of the trajectories were integrated into a single MD trajectory (SMT). The frequency distributions of the HSP90 RMSDs calculated using the SMT are plotted in Figure 2A. The RMSDs of the *apo*, W8V-, and W8S-bound HSP90s were respectively populated at the single peaks of 2.39, 2.01, and 2.01 Å, while the RMSD of the W8Y-bound HSP90 was centered at the bimodal positions of 2.01 and 2.89 Å. Compared to the *apo* HSP90, the binding of W8V and W8S weakened the structural fluctuation of HSP90; however, in HSP90, the presence of W8Y induced more obvious structural fluctuation. As shown in Figure 1C–E, the phenyl group of W8Y misses the alkyl group compared to W8V and W8S, which possibly leads to a more obvious structural fluctuation of HSP90. To examine the stability of W8Y, W8V, and W8S in the binding pocket of HSP90 after the equilibrium of the three inhibitor–HSP90 systems, the RMSDs of heavy atoms from three inhibitors were estimated. Moreover, their frequency distribution is displayed in Figure 2B. It was found that the RMSDs of W8Y, W8V, and W8S were distributed as 1.56, 1.63, and 0.65 Å, respectively, suggesting that three inhibitors were well preserved at the binding pocket of HSP90 throughout the MD simulations, especially for W8S.

To understand the effect of inhibitor binding on the structural flexibility of HSP90, root-mean-square fluctuations (RMSFs) of the Cα atoms in HSP90 were computed based on the SMT (Figure 2C). As a result, the binding of the three inhibitors exerts an important effect on the structural flexibility of HSP90. The structural domains with the most notable changes in RMSFs are highlighted in Figure S2. The binding of W8Y, W8V, and W8S significantly increased the RMSF of the D1 domain, implying that the presence of the three inhibitors strengthens the structural flexibility of D1 (Figure 2C and Figure S2). The binding of the three inhibitors decreased the RMSFs of two domains (D2 and D3), which shows that the binding of W8Y, W8V, and W8S weakens the structural flexibility of D2 and D3 and tends to make the structures of these two domains more rigid. Meanwhile, the results imply that three domains (D1, D2, and D3), which displayed alterations in RMSFs, are possibly involved in hot spots of inhibitor–HSP90 binding.

To further investigate the influence of inhibitor associations on the compactness of HSP90, the gyrations of HSP90 in four systems were computed using the SMT method. The frequency distributions of these gyrations are plotted in Figure 2D. The distribution shapes of the gyrations for the W8Y-, W8V-, and W8S-bound HSP90s move toward the right and have wider distributions compared to the *apo* HSP90, which implies that the presence of W8Y, W8V, and W8S in the pocket leads to looser structures in the HSP90 compared to the *apo* HSP90. In the previous RMSD analyses, the binding of the inhibitors had a significant effect on the HSP90 structures that was similar to the referenced conformations. This, in turn, affected the structural flexibility of HSP90.

In summary, the binding of the inhibitors significantly influences the structural fluctuations and flexibility of HSP90. In fact, the structural flexibility of HSP90 plays an important role in its functions [33]. The changes in flexibility and compactness of HSP90 caused by the binding of the inhibitors certainly generate significant impacts on the function of HSP90. The two previous reports that employed MD simulations indicated that inhibitor binding also induced changes in the structural flexibility of HSP90 [33,53], which supports our current results.

### 2.2. Changes in Dynamics Behavior of HSP90 Induced by Inhibitor Binding

**Figure 3:**
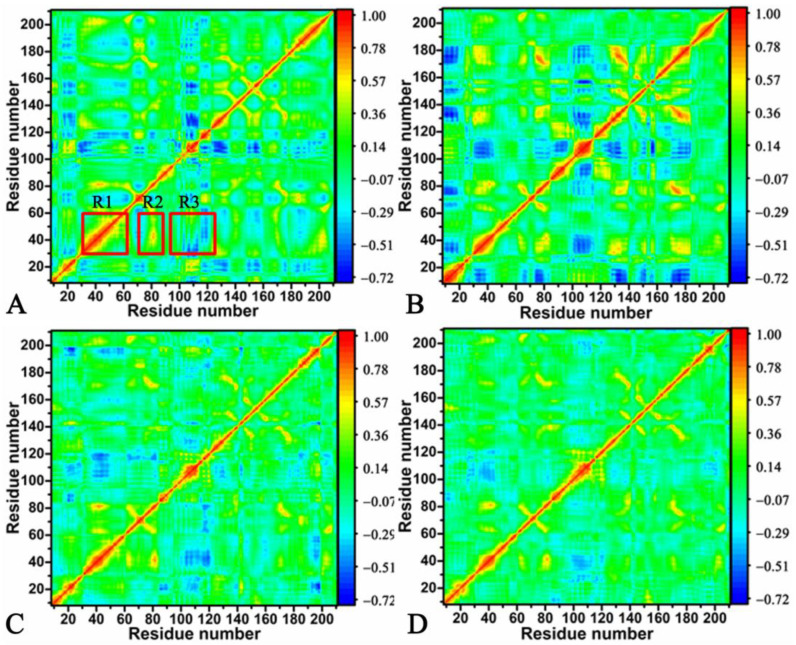
Dynamics cross-correlation maps calculated using the coordinates of the Cα atoms in HSP90: (**A**) the *apo* HSP90, (**B**) the W8Y-bound HSP90, (**C**) the W8V-bound HSP90, and (**D**) the W8S-bound HSP90. In this figure, the color bar represents the varying extents of the correlated motions between the structural domains.

To probe for alterations in the internal dynamics of HSP90 caused by the binding of inhibitors, dynamics cross-correlation maps (DCCMs) were estimated using the coordinates of the Cα atoms obtained from HSP90 (Figure 3). The extents of the correlated motions between the structural domains are represented by the color bar depicted in the right of the figures. As observed in Figure 3, inhibitor binding produces an effect on the motion modes of HSP90. In the *apo* HSP90, the R1 region generated strong positively correlated movements (PCMs) in relation to the D1 structure relative to itself (Figure 3A and Figure S2). The R2 region demonstrated strong PCMs in the D2 structure domain compared to the D1 structure domain, which is represented in red and yellow (Figure 3A and Figure S2). The R3 region characterized the strong anticorrelated motions (ACMs) between the D3 structure domain and the D1 structure (Figure 3A and Figure S2). Compared to the *apo* HSP90, the binding of W8Y, W8V, and W8S slightly weakened the PCM of the D1 structure relative to itself (Figure 3B–D). Meanwhile, the binding of the three inhibitors also slightly abated the PCMs between the D2 and D1 structure domains compared to the *apo* HSP90 (Figure 3B–D). The binding of W8Y strengthened the ACMs of the D3 structure relative to the D1 structure compared to the *apo* HSP90; however, the presence of W8V and W8S slightly weakened the ACMs of this region (Figure 3B–D). The aforementioned regions are involved in the binding sites of the HSP90 inhibitors. Thus, the changes in the motion modes of HSP90 certainly imply the exitance of alterations in the binding ability of HSP90 inhibitors.

To understand the impacts of inhibitor binding on the dynamics behavior of HSP90, PCA was performed on the SMT. The eigenvalues obtained from the PCA were used to characterize structural fluctuation along an eigenvector. The function of the eigenvalues as the eigenvector indexes is depicted in Figure S3. In general, the initial higher eigenvalues reflect the concerted motions of proteins in a subspace. The first 10 eigenvalues account for 84.04, 84.15, 77.10, and 80.41% of the total movement for the *apo* HSP90 and the W8Y-, W8V- and W8S-bound HSP90s, respectively (Figure S3). Compared to the *apo* HSP90, the first eigenvalue of the W8Y-, W8V-, and W8S-bound HSP90s is decreased, particularly for the W8V- and W8S-bound HSP90s, indicating that inhibitor binding inhibits the structural fluctuation of HSP90 along the first eigenvector.

**Figure 4:**
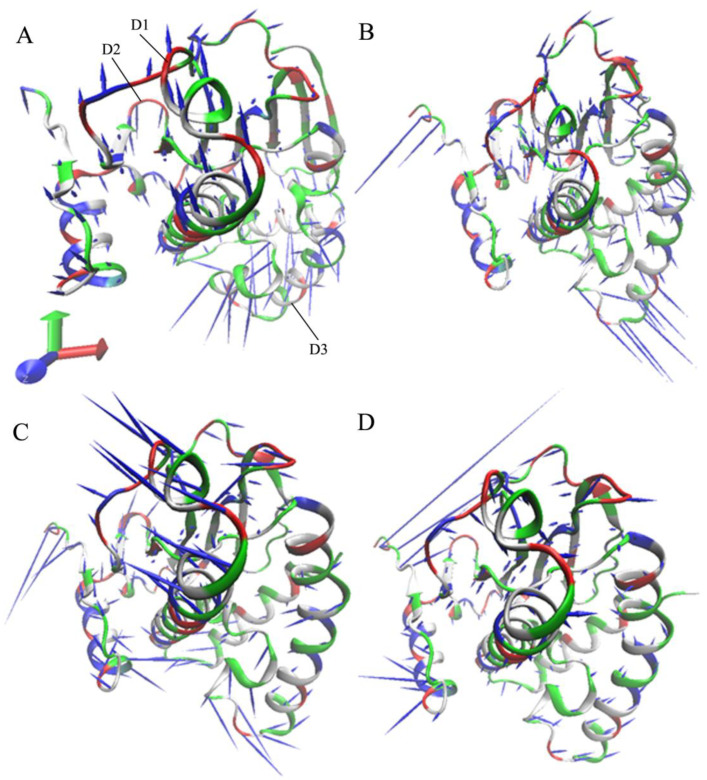
Concerted motions of HSP90 visualized using the first eigenvector: (**A**) the apo HSP90, (**B**) the W8Y-bound HSP90, (**C**) the W8V-bound HSP90, and (**D**) the W8S-bound HSP90. In this figure, HSP90 is displayed in cartoon modes.

To better explore the concerted motion of HSP90, the first eigenvector was visualized using the Visual Molecular Dynamics (VMD) program (Figure 4). It was observed that the presence of W8Y, W8V, and W8S affects the concerted movement of HSP90. Compared with the *apo* HSP90 (Figure 4A), the binding of W8Y and W8S both changed the tendency of the concerted movement of the structural domain (D1: residue 38–62) and weakened the fluctuation amplitude of the D1 domain (Figure 4B,D). The binding of W8V slightly altered the tendency of the concerted motions of the D1 structural domain. Meanwhile, it also strengthened the fluctuation amplitude of this domain (Figure 4C). Compared to the *apo* HSP90 (Figure 4A), the binding of W8Y, W8V, and W8S scarcely influenced the concerted motion of the structural domain D2 (residue 63–77) (Figure 4B–D). Similarly, compared to the *apo* HSP90, the binding of W8Y, W8V, and W8S significantly changed the tendency of the concerted movement of the D3 structural domain (residue 90–125). Conversely, the presence of W8Y and W8V did not inhibit the fluctuation amplitude of the D3 (Figure 4B,C); however, the binding of W8S evidently abated the fluctuation amplitude of the concerted motion of the D3 (Figure 4D).

**Figure 5:**
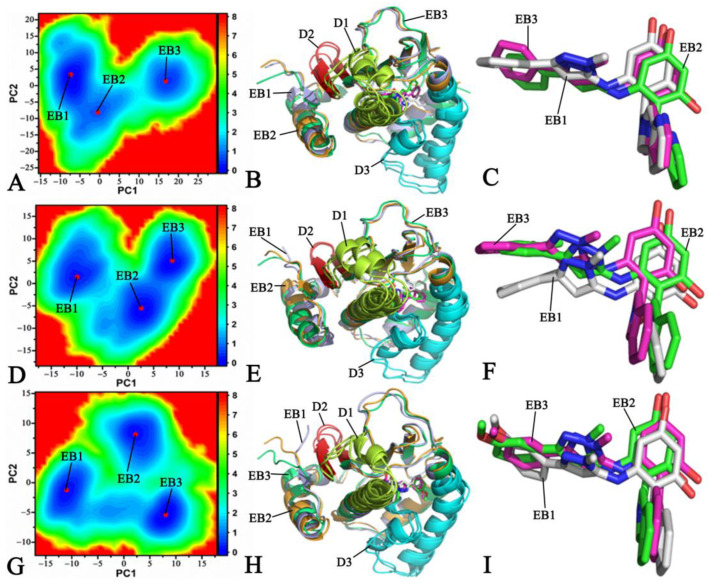
Free energy landscapes and the representative structures: (**A**) free energy landscape of the W8Y-bound HSP90, (**B**) structural superimpositions of the W8Y-bound HSP90 located at EB1-EB3, (**C**) structural alignment of W8Y falling into the EB1-EB3 range, (**D**) free energy landscape of the W8V-bound HSP90, (**E**) structural alignment of the W8V-bound HSP90 situated at EB1-EB3, (**F**) structural superimposition of W8V located at EB1-EB3, (**G**) free energy landscape of the W8S-bound HSP90, (**H**) superimposition of the W8S-bound HSP90, and (**I**) structural alignment of W8S trapped at EB1–EB3.

To reveal the energy source of the changes in the concerted motions caused by the binding of inhibitors, free energy landscapes (FELs) were built using the projections of the trajectory onto the first two principal components as reaction coordinates (RCs). FELs and information on the representative structures are displayed in Figure 5 and Figure S4. As shown in Figure 5 and Figure S4, the presence of W8Y, W8V, and W8S changes the free energy profiles of HSP90 and leads to a structural rearrangement of HSP90.

Regarding the *apo* HSP90, three independent MD simulations captured five energy basins that were respectively labeled as EB1, EB2, EB3, EB4, and EB5 (Figure S4A). This result implies that the structures of the *apo* HSP90 are mainly populated at five conformational subspaces. To further understand the structural difference between the *apo* HSP90 and the different energy states, five representative structures falling into the EB1-EB5 range were superimposed (Figure S4B). It was found that the D1, D2, and D3 structural domains generate obvious deviations, especially for D3. According to Figure 5A,D,G, the number of the energy basins of the W8Y-, W8V-, and W8S-bound HSP90s detected by the three independent MD simulations is three, labeled as EB1-EB3. Their number is less than that of the *apo* HSP90, which indicates that the binding of inhibitors leads to a conformational convergence of HSP90 and stabilizes the structures of HSP90. Compared to the *apo* HSP90, the binding of inhibitors results in better structural alignment (Figure 5B,E,H). Compared to the *apo* HSP90, the binding of W8Y and W8V induces more disordered states of the D1 structural domain (Figure 5B,E). Meanwhile, the binding of W8Y and W8S induces obvious deviations in the D2 structural domain (Figure 5B,H). Although HSP90 yields obvious conformational changes, the binding poses of the inhibitors do not generate deviations except for W8V.

Based on the above analyses, the binding of inhibitors alters correlated motions between the structural domains of HSP90 and affects the concerted motions of HSP90. At the same time, the presence of W8Y, W8V, and W8S induces less energy states than the *apo* HSP90, clarifying the energy basis with respect to the conformational changes in HSP90. The conformational changes in HSP90 exert significant influence on the binding of inhibitors to HSP90. The previous two studies also reported similar effects [53,56], reinforcing the results of our current work.

### 2.3. Binding Free Energy Calculations through MM-GBSA Method

To study the binding ability of inhibitors, the binding free energies of inhibitors to HSP90 were calculated using the molecular mechanics generalized Born surface area (MM-GBSA) method. To explore the effect of the different generalized Born (GB) model on the results, four GB models, discriminated by IGB = 1, IGB = 2, IGB = 5, and IGB = 66, were selected to compute binding free energies of inhibitors to HSP90. The parameters used in the four GB models are listed in Table 1, which includes two empirical parameters (γ and β) together with the radii types. The binding free energies and their components calculated using the MM-GBSA method are provided in Table 2.

As shown in Table 2, the binding free energies are divided into van der Waals interactions ($\Delta E_{vdW}$), electrostatic interactions ($\Delta E_{ele}$), polar solvation free energy ($\Delta G_{gb}$), non-polar solvation free energy ($\Delta G_{surf}$), and entropy contributions (−T∆S). Among the free energy components, $\Delta E_{vdW}$, $\Delta E_{ele}$, and $\Delta G_{surf}$ provide favorable contributions to the inhibitor–HSP90 binding, while $\Delta G_{gb}$ and −T∆S are unfavorable for inhibitor–HSP90 associations (Table 2). The sum of $\Delta E_{vdW}$ and $\Delta G_{surf}$ forms hydrophobic interactions of inhibitors with HSP90, which is advantageous for inhibitor–HSP90 associations. The sum of $\Delta E_{ele}$ and $\Delta G_{gb}$ constructs polar interactions of inhibitors with HSP90, which provides an unfavorable force for inhibitor–HSP90 binding. The sum of four components, $\Delta E_{vdW}$, $\Delta G_{surf}$, $\Delta E_{ele}$, and $\Delta G_{gb}$, forms the enthalpy contributions (∆H) to the binding of inhibitors to HSP90. As observed in Table 2, the GB models selected for the calculations of MM-GBSA only generate significant influences on polar solvation free energies, and the selection of the parameters γ and β exerts an impact on non-polar solvation free energies. Among the four GB models, the GB model of IGB = 5 leads to the weakest polar solvation free energies for all inhibitors, while that of IGB = 66 results in the strongest polar solvation free energies (Table 2). Correspondingly, the GB model of IGB = 5 generates the strongest enthalpy contributions to inhibitor–HSP90 binding, while that of IGB = 66 produces the weakest enthalpy contributions to inhibitor–HSP90 associations. As a result, the selection of the GB models is vital in the calculation of binding free energies.

Among the four GB models, the binding free energies of W8V and W8S to HSP90 calculated using a GB model of IGB = 66 are the closest to the experimental values, while that of W8V and W8S to HSP90, computed using a GB model of IGB = 5, mostly deviate from the experimental results. Despite this, the energies computed using a GB model of IGB = 66 are still overestimated by one order of magnitude than that of the experimental energies, an outcome that has also been observed in previous works [35,38]. Thus, this result does not affect our rational explanation of the binding ability of inhibitors to HSP90. On the other hand, the rankings of the binding free energies of W8V and W8S to HSP90 in the four GB models also agree with those obtained from the experimental values, which suggests that our current results are rational. Thus, the results calculated using a GB model of IGB = 66 were used to evaluate the binding difference of W8Y, W8V, and W8S to HSP90. The electrostatic interactions of W8V and W8S with HSP90 were respectively strengthened by 13.56 and 12.71 kcal/mol compared to that of W8Y with HSP90; however, the unfavorable polar solvation free energies of the W8V– and W8S–HSP90 complexes increased by 9.62 and 12.27 kcal/mol relative to the W8Y–HSP90 complex. As a result, the polar interactions of W8V with HSP90 increased by 3.94 kcal/mol relative to W8Y with HSP90, while the polar interaction of W8S with HSP90 was weakened by 0.44 kcal/mol. The hydrophobic interactions of W8V and W8S with HSP90 were strengthened by 0.42 and 1.96 kcal/mol compared to W8Y with HSP90, respectively. Overall, the enthalpy contributions to W8V– and W8S–HSP90 binding were respectively enhanced by 4.36 and 2.4 kcal/mol compared with W8Y–HSP90. Thus, although structural difference among the three inhibitors is small, their respective binding abilities to HSP90 vary significantly in our calculations, which is likely due to the conformational changes caused by their binding. It was found that van der Waals interactions are much bigger than non-polar solvation free energies. Thus, van der Waals interactions provide the greatest contribution to inhibitor binding, which is in good agreement with previous reports [53,67]. Based on this result, optimization using the van der Waals interactions of inhibitors with HSP90 is a key element in the successful design of efficient inhibitors toward HSP90.

### 2.4. Interaction Network of Inhibitors with HSP90

**Figure 6:**
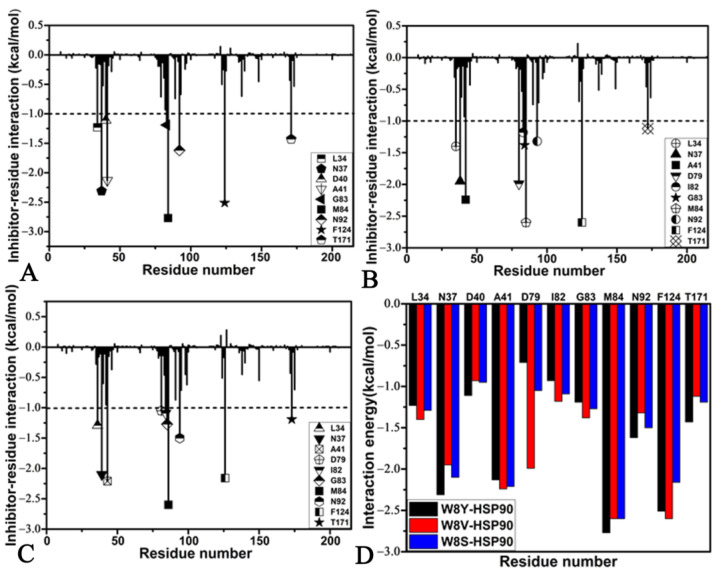
Inhibitor–residue interaction spectrum: (**A**) W8Y, (**B**) W8V, (**C**) W8S, and (**D**) key residues in the binding of W8Y, W8V, and W8S to HSP90. In this figure, residues that provided contributions stronger than −1.0 kcal/mol are labeled.

**Figure 7:**
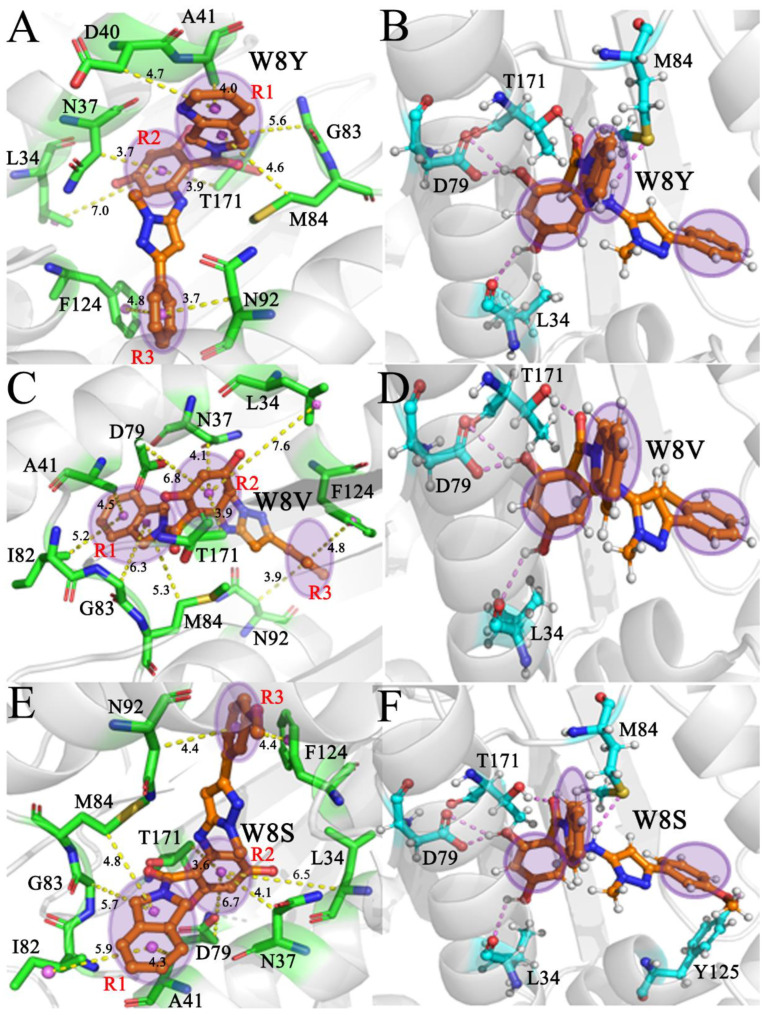
Hydrophobic interactions and HBIs of inhibitors with residues in HSP90: (**A**,**B**) corresponding to hydrophobic interactions and HBIs of W8Y with HSP90, respectively, (**C**,**D**) indicating hydrophobic interactions of HBIs of W8V with HSP90, respectively, and (**E**,**F**) representing hydrophobic interactions and HBIs of W8S with HSP90, respectively.

To examine the roles of separate residues in inhibitor bindings, a residue-based free energy decomposition approach was employed to estimate interactions of W8Y, W8V, and W8S with separate residues from HSP90 (Figure 6A–C). The contributions of the key residues are highlighted in Figure 6D, and significant components are provided in Table 3. Hydrogen-bonding interactions (HBIs) between inhibitors and HSP90 were analyzed through the CPPTRAJ module in Amber 20, and the corresponding results are given in Table 4. Regarding inhibitor–residue interactions, including hydrophobic interactions and HBIs, the obtained structural information is displayed in Figure 7 using the lowest energy structures collected from the MD simulations.

Regarding the W8Y–HSP90 complex, the interactions of W8Y with nine residues in HSP90 are stronger than −1.0 kcal/mol, and these residues include L34, N37, D40, A41, G83, M84, N92, F124, and T171 (Figure 6A,D). Structurally, the alkyls or carbon atoms of residues D40, A41, G83, and M84 are located near the hydrophobic ring R1 of W8Y; thus, the CH–π interactions are easily formed between them (Figure 7A). Moreover, the M84 residue produces an HBI with W8Y with an occupancy of 33.3% (Table 4 and Figure 7B). Overall, D40, A41, G83, and M84 provide energy contributions of −1.11, −2.13, −2.77, and −1.62 kcal/mol for the binding of W8Y, respectively (Figure 6A,D). Among these four residues, the sum of the electrostatic interactions and polar solvation free energy, as well as the non-polar solvation free energy, only contribute a weak force to the binding of W8Y to these four residues (Table 3). Thus, the interactions of W8Y with D40, A41, G83, and M84 mainly stem from van der Walls interactions. The alkyls or carbon atoms of residues L34, N37, and T171 are adjacent to the hydrophobic ring R2 of W8Y; moreover, they tend to generate the CH–π interactions (Figure 7A). Meanwhile, W8Y yields two HBIs with residues L34 and T171, with an occupancy rate higher than 67.7% (Table 4 and Figure 7B). Based on Figure 6A,D, the interaction energies of L34, N37, and T171 with W8Y are −1.23, −2.31, and −1.43 kcal/mol, respectively, which is favorable for the W8Y–HSP90 binding. According to Table 3, the energy contributions of N37 and T171 mostly arise from van der Waals interactions, while that of L34 is mainly contributed by the sum of the van der Waals interaction and electrostatic interaction. As observed in Figure 6A,D, the residues N92 and F124 respectively provide energy contributions of −1.62 and −2.51 kcal/mol for the binding of W8Y, which structurally agrees with the π–π interaction of the phenyl group in F124 and the CH–π interaction of the CH group in N92 with the hydrophobic ring R3 in W8Y (Figure 7B). Table 3 shows that the interactions of W8Y with N92 and F124 are almost derived from the contributions of van der Waals interactions. In addition, the D79 carbonyl group forms two HBIs with W8Y, and the occupancy rate of these two HBIs is higher than 49.8% (Table 4 and Figure 7B); however, they only provide energy contributions of −0.71 kcal/mol for the binding of W8Y (Table 3).

In the case of the W8V–HSP90 complex, ten residues, including L34, N37, A41, D79, I82, G83, M84, N92, F124, and T171, provide energy contributions stronger than −1.0 kcal/mol in the association of W8V with HSP90 (Figure 6B,D). As shown in Figure 7C, the alkyls or carbon atoms of four residues (A41, I82, G83, and M84) are next to the hydrophobic ring R1 of W8V, which leads to the CH–π interactions between them. The interactions of A41, I82, G83, and M84 with W8V are −2.24, −1.18, −1.38, and −2.6 kcal/mol, respectively (Figure 6B,D). The interaction energies of I82 and M84 with W8V mostly stem from van der Walls interactions, while that of A41 and G83 come from the sum of van der Waals and polar interactions (Table 3). The alkyls or carbon atoms of residues L34, N37, and T171 are situated near the hydrophobic ring R2 of W8V, which results in the CH–π interactions between them (Figure 7C). Additionally, a hydrogen bond with an occupancy of 68.4% is detected between L34 and W8V (Figure 7D and Table 4). In these structures, the carbonyl group of D79 is next to the R2 of W8V; moreover, they can easily form a weak CH–O interaction between them. Meanwhile, the carbonyl of D79 also generates two hydrogen bonds with W8V, and their occupancy is higher than 62.1%. As a result, L34, N37, D79, and T171 contribute interaction energies of −1.4, −1.95, −1.99, and −1.12 kcal/mol to the W8V–HSP90 binding process (Figure 6B,D). As indicated in Table 3, the energy contributions of N37 and T171 mainly arise from van der Waals interactions, the energy contribution of D79 is provided by electrostatic interaction, and that of L34 mostly originates from the sum of van der Waals interactions and electrostatic interactions. According to Figure 7C, the phenyl group of F124 forms a π–π interaction with the hydrophobic ring R3 of W8V, and the CH group of N92 produces a CH–π interaction with the R3 of W8V (Figure 7C). The interaction energies of N92 and F124 with W8V are −1.32 and −2.60 kcal/mol (Figure 6B,D); moreover, their energy contributions almost stem from van der Waals interactions (Table 3).

Concerning the W8S–HSP90 complex, W8S shares similar interaction modes to W8Y and W8V. Although the interaction of W8S with D40 is weaker than that of W8Y with D40, the interactions of W8S with A41 and I82 are stronger than that of W8Y with these two residues (Figure 6C,D). The hot interaction spots of W8S with HSP90 are the same as those of W8V. (Figure 6C,D). Based on the structural information shown in Figure 7E,F, hydrophobic interactions and the HBIs of W8S with HSP90 are similar to that of W8V (Figure 6C,D and Table 4). Based on Table 3, the energy contributions of A41 and G83 are mostly provided by the sum of van der Waals and polar interactions, while that of I82 and M84 mainly stem from van der Waals interactions. Meanwhile, the favorable energy contributions of N37 and T171 mainly arise from van der Waals interactions with W8S, the one of L34 is contributed by the sum of van der Waals and electrostatic interactions, and the one of D79 almost completely comes from electrostatic interactions (Table 3). In addition, the energy contributions of N92 and F124 to the W8S–HSP90 binding process almost originates from the van der Waals interactions (Table 3). Although the residue Y125 forms a hydrogen bond with W8S and its occupancy is 35.7%, this residue only provides an unfavorable contribution of 0.28 kcal/mol (Figure 6C), which is possibly due to the repulsive electrostatic Interaction between the oxygen atoms of Y125 and the R3 of W8S.

**Figure 8:**
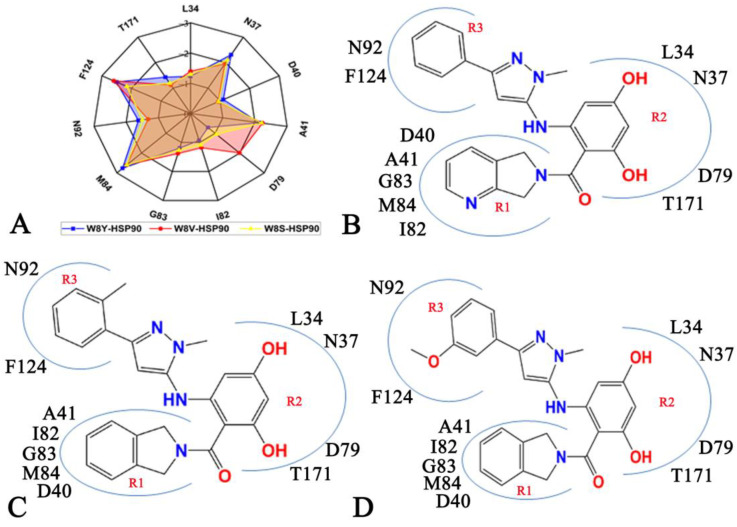
Hot spots of inhibitor–HSP90 binding: (**A**) radar representations of residues located at hot spots of inhibitor–HSP90 binding, (**B**) W8Y, (**C**) W8V, and (**D**) W8S.

As in Figure 6A–C, the favorable interactions of W8Y, W8V, and W8S with HSP90 are highlighted in Figure 8A. Residues D40, A41, I82, G83, and M84 interact with the hydrophobic ring R1 of three inhibitors (Figure 8C,D). Thus, these five residues are identified as the first hot spot of the inhibitor–HSP90 interactions. The L34, N37, D79, and T171 residues produce interactions with the hydrophobic ring R2 of three inhibitors (Figure 8C,D); hence, these four residues form the second hot spot of the inhibitor–HSP90 binding process. The N92 and F124 residues yield interactions with the hydrophobic ring R3 of three current inhibitors; as a result, N92 and F124 build the third hot spot of the inhibitor–HSP90 associations. Previous experimental and calculated works have also reported similar interactions between inhibitors and HSP90, which are fundamentally consistent with our current results [11,67]. Based these analyses, it is important to rationally optimize the interactions of these three hot spots with inhibitors to successfully design efficient drugs related to HSP90.

## 3. Theory and Methods

### 3.1. System Preparations

The initial structures of the *apo* HSP90 and HSP90 that were bound by three inhibitors (W8Y, W8V, and W8S) were extracted from the protein data bank (PDB): the PDB entries 7K9R, 7K9U, 7K9V, and 7K9W respectively correspond to the *apo*, W8Y-, W8V-, and W8S-bound HSP90s [11]. Due to differences in the residue sequence, residues 8–211 of HSP90 were used to construct four starting models. All water molecules in the crystal structures were kept in the initial model. The protonated states of residues in HSP90 were checked using H++ 3.0 [68] and the protonation states of residues were rationally set. The missing hydrogen atoms were added to their corresponding heavy atoms using the Leap module in Amber 20 [69,70]. The simulation parameters of HSP90 were obtained from the Amber ff19SB force field [71]. Three small-molecule inhibitors (W8Y, W8V, and W8S) were optimized using the semi-empirical AM1 approach; then, the atomic bond charge correction (BCC) charges [72,73] of W8Y, W8V, and W8S were produced using the Antechamber module [74] in Amber 20. The general AMBER force field (GAFF2) was applied to generate the force field parameters of three inhibitors, W8Y, W8V, and W8S [75,76]. The *apo* HSP90 and three inhibitor–HSP90 complexes involved in this study were immersed in a truncated octahedron water box, with a distance of at least 12 Å between the complex and the boundary of the water box, in which the force field parameters of the water molecules were obtained from the TIP3P water model [77]. The appropriate number of sodium ions (Na^+^) was placed in the water box in a 0.15 M NaCl salt environment to neutralize the simulation systems, from which the parameters of the Na^+^ and Cl^−^ ions were obtained, in accordance with the studies of Joung et al. [78,79].

### 3.2. Multiple Independent All-Atom Molecular Dynamic (AAMD) Simulations

Some high-energy contacts between atoms were possibly formed during the initial process of the four simulated systems, which likely resulted in the instability of the systems. To address this issue, two-step energy minimizations were executed before a real MD simulation, which included a 50,000-step steepest descent optimization procedure and a 50,000-step conjugate gradient one. Then, the optimized systems were subjected to a slow heating process from 0 to 300 k in 1 ns in the canonical ensemble (NVT), in which all heavy atoms of the four systems were constrained in a weak harmonic restriction of 2 kcalmol^−1^·Å^2^. After that, a 2-ns equilibrium process was performed on four systems at 300 K under the isothermal−isobaric ensemble (NPT) to further optimize the systems. Finally, 600-ns MD simulations were performed on each system to deeply relax the systems. The aforementioned simulation processes were repeatedly twice; in each running, the initial atomic velocities were assigned with the Maxwell distribution. As a result, three independent MD simulations were realized. During all simulations, the Langevin thermostat [80] was used to control the system temperature, in which the collision frequency was set as 2 ps^−1^. The shake algorithm [81] was employed to restrict all chemical bonds containing hydrogen atoms. The long-range electrostatic interactions were computed through the particle mesh Ewald algorithm [82] with a cutoff value of 9 Å. This cutoff was also adopted for the calculation of van der Waals interactions. The equilibrium parts of three independent MD trajectories were joined into a single MD trajectory (SMT) to make the postprocessing analysis more convenient. In this study, all simulations were conducted by employing the pmemd.cuda program in Amber 20 [83,84].

### 3.3. Principal Component Analysis and Dynamics Cross-Correlation Maps

PCA can help us better predict the collective motions of structure domains from HSP90. In this work, PCA was employed to decipher how the presence of inhibitors affects the collective motions of HSP90. PCA was realized by performing diagonalization on a covariance matrix C constructed using the coordinates of the Cα atoms from HSP90 using the following equation

(1) $$C=<\left(q_{i}-<q_{i}>\right){\left(q_{j}-<q_{j}>\right)}^{T}>$$

where the terms $q_{i}$ and $q_{j}$ indicate the Cartesian coordinates of the *i*th and *j*th Cα atoms from HSP90, respectively, while the terms $<q_{i}>$ and $<q_{j}>$ denote their averaged positions on conformational ensembles kept at the SAT. The average is usually calculated by aligning the SMT with a referenced structure to delete overall translations and rotations using a least-square fit procedure [85]. The eigenvalues and eigenvectors arising from the PCA are used to describe the fluctuation amplitude along an eigenvector and to collective motions of the structural domains, respectively.

Dynamics cross-correlation maps (DCCMs) are usually applied to characterize the internal dynamics of proteins. In this work, DCCMs were calculated using Equation (2) to reveal the effect of inhibitor bindings on correlated motions between residues of HSP90.

(2) $$C_{ij}=\frac{<\Delta r_{i}\;\cdot \;\Delta r_{j}>}{{\left(<\Delta r_{i}^{2}><\Delta r_{j}^{2}>\right)}^{1/2}}$$

where the terms $\Delta r_{i}$ and $\Delta r_{j}$ respectively reflect the displacement of the Cαatoms *i* and *j* relative to their corresponding averaged positions. The angle brackets imply ensemble averages on the snapshots saved using the SAT. The element values ($C_{ij}$) of the DCCMs are located using a range of −1 to 1. The positive and negative $C_{ij}\;$values respectively represent the positively correlated movements (PCMs) and the anticorrelated motions (ACMs) between the Cα atoms *i* and *j*. The color-coded bars are utilized to represent the extent of the correlated motions between the residues of HSP90. The module CPPTRAJ [86] in Amber 20 was also utilized to realize the PCA and computations of DCCMs.

### 3.4. Calculations of MM-GBSA

The molecular mechanism Poisson Boltzmann surface area (MM-PBSA) and MM-GBSA methods are two fast tools that enable the prediction of the binding free energies of inhibitors to proteins. Hou’s group performed a comparison of the performance of MM-PBSA and MM-GBSA using a series of proteins [35,46]. Based on their results, we used the MM-GBSA method to calculate inhibitor–HSP90 binding free energies. To obtain the current calculations, 500 snapshots were taken using the SMT to estimate binding free energies. Due to the extensive amount of time required for the entropy calculation, 100 snapshots stemming from the abovementioned 500 snapshots were adopted to compute the entropy contributions to the inhibitor–HSP90 associations. In this study, the binding free energies ($\Delta G_{bind}$) were divided into five components as expressed in Equation (3).

(3) $$\Delta G_{bind}=\Delta E_{ele}+\Delta E_{vdW}+\Delta G_{pol}+\Delta G_{nonpol}-T\Delta S$$

where $\Delta E_{ele}$ and $\Delta E_{vdW}$ are electrostatic interactions and van der Waals interactions are calculated using the molecular force field ff19SB, respectively. The two components $\Delta G_{pol}$ and $\Delta G_{nonpol}$ are polar solvation free energies and non-polar solvation free energies, respectively, in which the former is solved using the generalized Born (GB) model [87] and the latter is computed using an empirical equation $\Delta G_{nonpol}=\gamma \times \Delta SASA+\beta$, in which ΔSASA indicates the solvent-accessible surface area. The component −TΔS represents the contribution of the entropic change to binding free energies, which was calculated using the mmpbsa_py_nabnmode program in Amber 20 [88]. In this work, we selected different GB models that respectively indicated IGB = 1, 2, 5, and 66 [87,89] to calculate the enthalpy changes so that we could evaluate the effects of different GB models on the calculations of binding free energies. The type of GB models and the corresponding parameters, including radii types (γ and β), are provided in Table 1.

## 4. Conclusions

The exploration of the conformational changes of HSP90 caused by inhibitor binding and inhibitor–HSP90 identification sites is of high importance to understand the target roles of HSP90 in drug designs created to combat human diseases. In our current study, three independent MD simulations, each running for 600 ns, were conducted on the *apo* HSP90 and W8Y-, W8V-, and W8S-bound HSP90s. RMSFs and DCCMs were used to calculate MD trajectories, the results of which indicate that inhibitor binding highly impacts the structural flexibility and correlated motions of HSP90. PCA and FELs constructed using the principal components PC1 and PC2 suggest that the presence of inhibitors not only changes the dynamics behavior of HSP90 but also induces alterations in the free energy profiles and conformational rearrangement of HSP90. Our analysis of different GB models verified that the selection of GB models and empirical parameters affect the predictions of binding free energies and showed that van der Waals interactions are the main forces responsible for inhibitor–HSP90 binding. These analyses of interaction networks suggest that residues L34, N37, D40, A41, D79, I82, G83, M84, F124, and T171 can be used as targeting sites for the design of potent inhibitors of HSP90.

## Figures and Tables

**Table 1:** The parameters used in MM-GBSA calculations with different generalized Born model.

Parameters	IGB = 1	IGB = 2	IGB = 5	IGB = 66
^a^ γ	0.0072	0.005	0.005	0.005
^a^ β	0.00	0.00	0.00	0.00
^b^ radii	mbondi	mbondi2	mbondi2	bondi

^a^ Two empirical parameters used calculations of MM-GBSA. ^b^ Radius type used in selections of GB model, including mbondi, mbondi2, and bondi.

**Table 2:** Binding free energies calculated using the MM-GBSA method with different GB models ^a^.

Energy	W8Y				W8V				W8S			
	IGB = 1	IGB = 2	IGB = 5	IGB = 66	IGB = 1	IGB = 2	IGB = 5	IGB = 66	IGB = 1	IGB = 2	IGB = 5	IGB = 66
$\Delta E_{ele}$	−35.72	−35.72	−35.72	−35.72	−49.28	−49.28	−49.28	−49.28	−48.43	−48.43	−48.43	−48.43
$\Delta E_{vdW}$	−53.18	−53.18	−53.18	−53.18	−53.49	−53.49	−53.49	−53.49	−54.80	−54.80	−54.80	−54.80
$\Delta G_{gb}$	50.21	46.74	9.99	54.55	60.89	57.70	13.85	64.17	61.90	58.81	18.67	66.82
$\Delta G_{surf}$	−6.76	−4.70	−4.70	−4.70	−6.93	−4.81	−4.81	−4.81	−7.25	−5.04	−5.04	−5.04
^b^ $\Delta G_{pol}$	14.49	11.02	−25.73	18.83	11.61	8.42	−35.43	14.89	13.47	10.38	−29.76	18.39
^c^ $\Delta G_{hydro}$	−59.94	−57.88	−57.88	−57.88	−60.42	−58.30	−58.30	−58.30	−62.05	−59.84	−59.84	−59.84
^d^ ΔH	−45.45	−46.86	−83.61	−39.05	−48.81	−49.88	−93.73	−43.41	−48.58	−49.46	−89.60	−41.45
−TΔS	23.27				23.05				22.56			
$\Delta G_{bind}$	−22.18	−23.58	−60.33	−15.78	−25.78	−26.84	−70.70	−20.38	−26.03	−26.90	−67.05	−18.89
^e^ $\Delta G_{exp}$	^f^ --				−9.83				−8.96			

^a^ All free energy components are scaled in kcal/mol. ^b^ $\Delta G_{pol}=\Delta E_{ele}+\Delta G_{gb}$, which is used to describe polar interactions of inhibitors with HSP90. ^c^ $\Delta G_{hydro}=\Delta E_{vdW}+\Delta G_{surf}$, which is utilized to signify hydrophobic interactions of inhibitors with HSP90. ^d^ $\Delta H=\Delta G_{pol}+\Delta G_{hydro}$, which is adopted to indicate the enthalpy effect during bindings of inhibitors to HSP90. ^e^ $\Delta G_{exp}$ is obtained by using ΔG=−RTlnEC50 with an experimental value of EC50. ^f^ The experimental EC50 value is unavailable. In this table, the mean errors of $\Delta E_{ele},\;\Delta G_{gb}$, and $\Delta G_{pol}$ are respectively located in the following ranges: 0.51 to 0.71, 0.64 to 0.82, and 0.55 to 0.74 kcal/mol; the mean errors of $\Delta E_{vdW}$, $\Delta G_{surf}$, and $\Delta G_{hydro}$ are respectively located in the following ranges: 0.25 to 0.31, 0.01 to 0.02, and 0.13 to 0.17 kcal/mol; the mean errors of ΔH, −TΔS, and $\Delta G_{bind}$ are respectively located in the following ranges: 0.34 to 0.46, 0.33 to 0.36, and 0.35 to 0.41 kcal/mol.

**Table 3:** Separate components of inhibitor–residue interactions calculated using the MM-GBSA method.

Inhibitor	Residue	$\Delta Vdw_{tot}$	$\Delta Ele_{tot}$	$\Delta GB_{tot}$	$\Delta Surf_{tot}$	ΔG
W8Y–HSP90	L34	−0.72	−2.08	1.58	−0.01	−1.23
	N37	−2.72	0.35	0.27	−0.22	−2.31
	D40	−1.00	−0.59	0.58	−0.10	−1.11
	A41	−1.78	−0.10	−0.13	−0.11	−2.13
	D79	0.90	−9.45	7.86	−0.02	−0.71
	I82	−0.82	0.00	−0.06	−0.06	−0.93
	G83	−0.89	−2.23	1.95	−0.03	−1.19
	M84	−2.42	−0.65	0.52	−0.21	−2.77
	N92	−1.73	−0.46	0.72	−0.15	−1.62
	F124	−2.71	−0.60	1.01	−0.21	−2.51
	T171	−1.01	−3.94	3.61	−0.10	−1.43
W8V–HSP90	L34	−0.70	−2.75	2.07	−0.02	−1.40
	N37	−2.81	0.14	0.95	−0.23	−1.95
	D40	−0.98	−1.77	1.91	−0.10	−0.93
	A41	−1.70	0.11	−0.54	−0.11	−2.24
	D79	0.98	−13.30	10.34	−0.02	−1.99
	I82	−0.89	−0.19	−0.03	−0.07	−1.18
	G83	−0.95	−2.06	1.66	−0.03	−1.38
	M84	−2.50	−0.46	0.61	−0.24	−2.60
	N92	−1.54	−0.42	0.77	−0.12	−1.32
	F124	−2.45	−1.15	1.20	−0.20	−2.60
	T171	−1.13	−3.50	3.62	−0.11	−1.12
W8S–HSP90	L34	−0.68	−2.49	1.91	−0.03	−1.29
	N37	−2.89	−0.27	1.30	−0.25	−2.10
	A41	−1.70	0.05	−0.45	−0.11	−2.21
	D79	0.83	−11.16	9.31	−0.02	−1.05
	I82	−0.86	−0.12	−0.04	−0.06	−1.09
	G83	−0.94	−2.01	1.72	−0.03	−1.27
	M84	−2.40	−0.54	0.58	−0.24	−2.60
	N92	−1.78	−0.58	0.99	−0.13	−1.50
	F124	−2.26	−0.58	0.88	−0.20	−2.16
	T171	−1.00	−3.79	3.70	−0.11	−1.19

**Table 4:** HBIs of inhibitors with residues in HSP90 analyzed using the CPPTRAJ module.

Compound	^a^ Hydrogen Bonds	Distance (Å)	Angle (°)	^b^ Occupancy (%)
W8Y–HSP90	W8Y-O17∙∙∙T171-HG1-OG1	2.82	160.71	99.44
	D79-OD1∙∙∙W8Y-H9-O14	2.69	161.62	73.71
	L34-O∙∙∙W8Y-H21-O11	3.04	139.04	67.66
	D79-OD2∙∙∙W8Y-H9-O14	2.91	151.67	49.82
	M84-SD∙∙∙W8Y-H20-N07	3.33	146.72	33.34
W8V–HSP90	W8V-O17∙∙∙T171-HG1-OG1	2.77	156.21	97.56
	D79-OD1∙∙∙W8V-H24-O14	2.79	160.14	76.63
	L34-O∙∙∙W8V-H23-O11	3.04	141.22	68.38
	D79-OD2∙∙∙W8V-H24-O14	2.85	152.23	62.14
W8S–HSP90	W8S-O24∙∙∙T171-HG1-OG1	2.70	160.01	99.16
	W8S-O02∙∙∙Y125-HH-OH	3.22	158.06	35.74
	D79-OD2∙∙∙W8S-H24-O21	2.71	161.13	76.27
	L34-O∙∙∙W8S-H23-O18	3.03	141.36	64.42
	D79-OD1∙∙∙W8S-H24-O21	2.93	152.34	50.48
	M84-SD∙∙∙W8S-H22-N14	3.33	146.02	35.33

^a^ Hydrogen-bonding interactions are determined using an acceptor∙∙∙donor distance of <3.5 Å and an acceptor∙∙∙H-donor angle of >120°. ^b^ Occupancy (%) is defined as the percentage of the simulation time that a specific hydrogen bond exists.

## Data Availability

Not applicable.

## Supplementary Materials

The following supporting information can be downloaded at: https://www.mdpi.com/article/10.3390/molecules28124792/s1, Figure S1. Root-mean-square deviations (RMSDs) of backbone atoms in HSP90 in three independent simulations; Figure S2. Structural domains of HSP90 with significant changes in RMSF results, in which D1, D2, and D3 are represented by different domains with respective colors; Figure S3. The function of eigenvalues as eigenvector indexes from principal component analysis, which is used to describe structural fluctuations of HSP90 along the eigenvectors; Figure S4. Free energy landscapes and the representative structures of the *apo* HSP90.

## Sample Availability

Samples of the compounds are not available from the authors.
