# Supplementary material for: Binding Mechanism of Inhibitors to Heat Shock Protein 90 Investigated by Multiple Independent Molecular Dynamics Simulations and Prediction of Binding Free Energy
Source: Molecules. 2023 Jun 15;28(12):4792. doi: 10.3390/molecules28124792 (PMC10304427; doi:10.3390/molecules28124792)
Supplement: Supplementary file 1 [file molecules-28-04792-s001.zip › molecules-2391028-supplementary.pdf]

molecules

Article

Binding Mechanism of Inhibitors to Heat Shock Protein 90 Investigated by Multiple Independent Molecular Dynamics Simulations and Prediction of Binding Free Energy

Fen Yang [1], Yiwen Wang [1,2], Dongliang Yan [1,3], Zhongtao Liu [1], Benzheng Wei [4], Jianzhong Chen [3,*] and Weikai He [2,*]

1 School of Information Science and Electrical Engineering, Shandong Jiaotong University, Jinan 250357, China; fen_yang0223@163.com (F.Y.)
2 School of Aeronautics, Shandong Jiaotong University, Jinan 250357, China
3 School of Science, Shandong Jiaotong University, Jinan 250357, China
4 Center for Medical Artificial Intelligence, Shandong University of Traditional Chinese Medicine, Qingdao 266112, China
* Correspondence: chenjianzhong1970@163.com or jzchen@sdjtu.edu.cn (J.C.); 214035@sdjtu.edu.cn (W.H.)

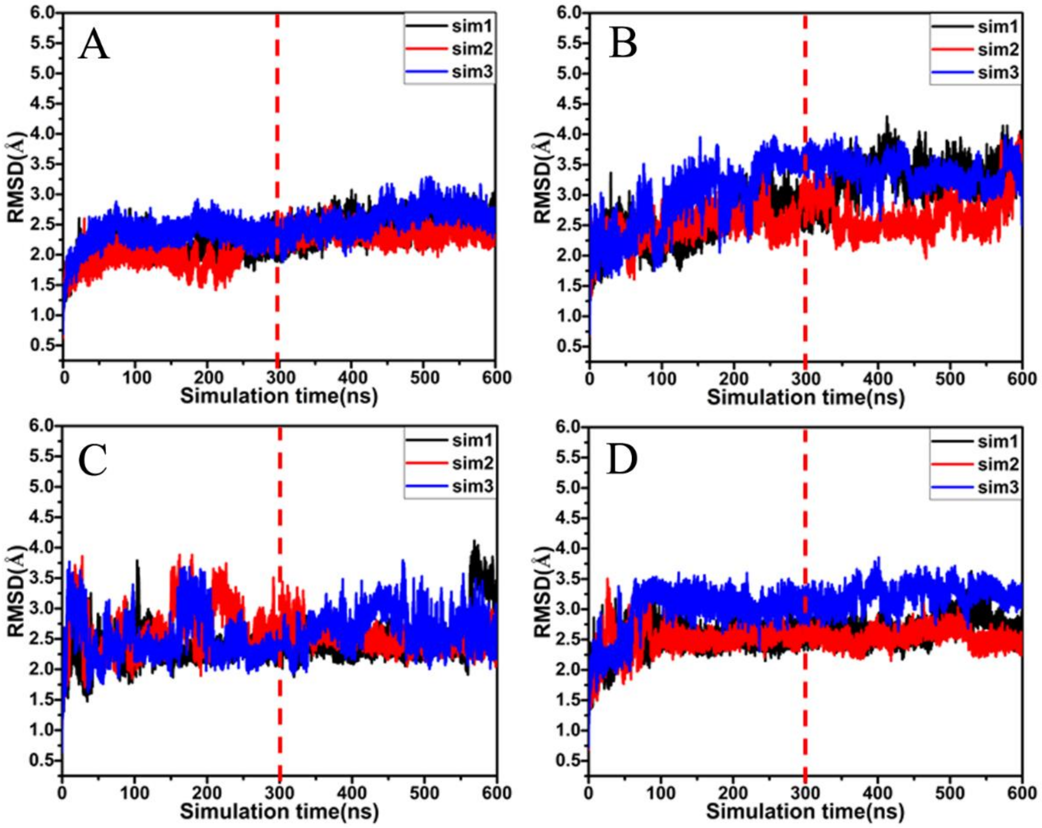

Figure S1. Root-mean-square deviations (RMSDs) of backbone atoms in HSP90 in three independent simulations: (A) the *apo* HSP90, (B) the W8Y-bound HSP90, (C) the W8V-bound HSP90 and (D) the W8S-bound HSP90.

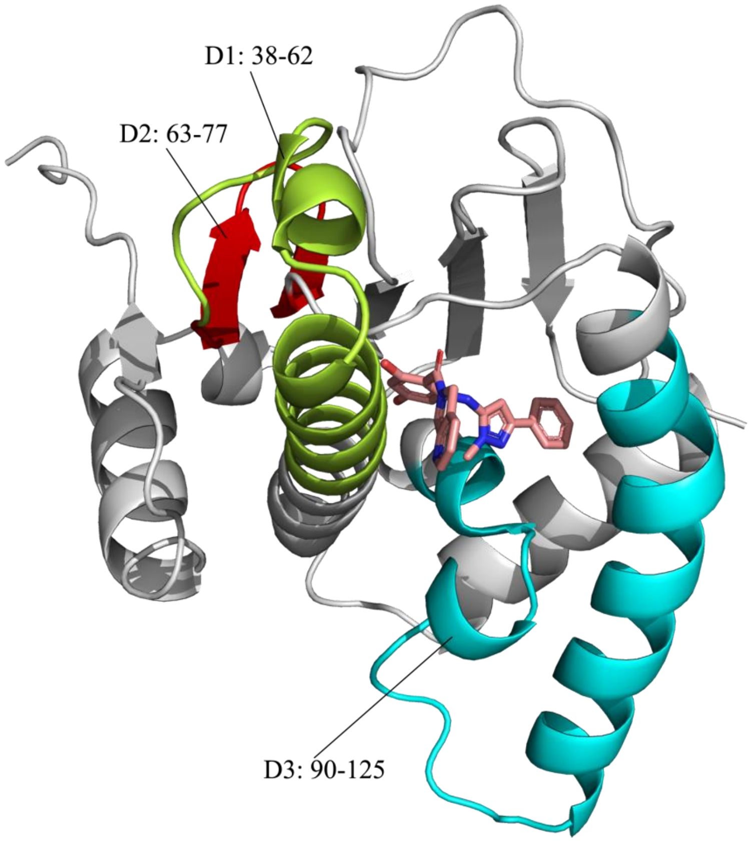

Figure S2. Structural domains of HSP90 with obvious changes of RMSF, in which the D1, D2 and D3 display different domains with colors.

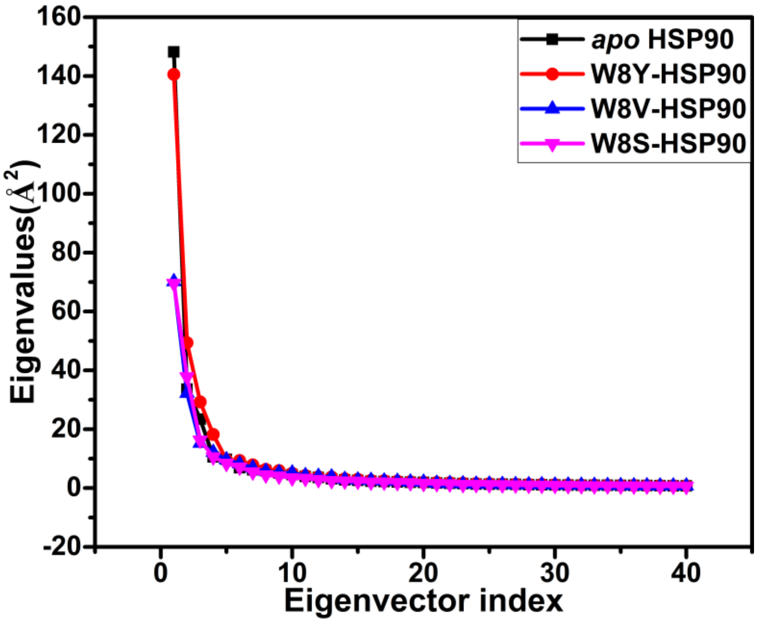

Figure S3. The function of eigenvalues as eigenvector indexes from principal component analysis, which is used to describe structural fluctuations of HSP90 along the eigenvectors.

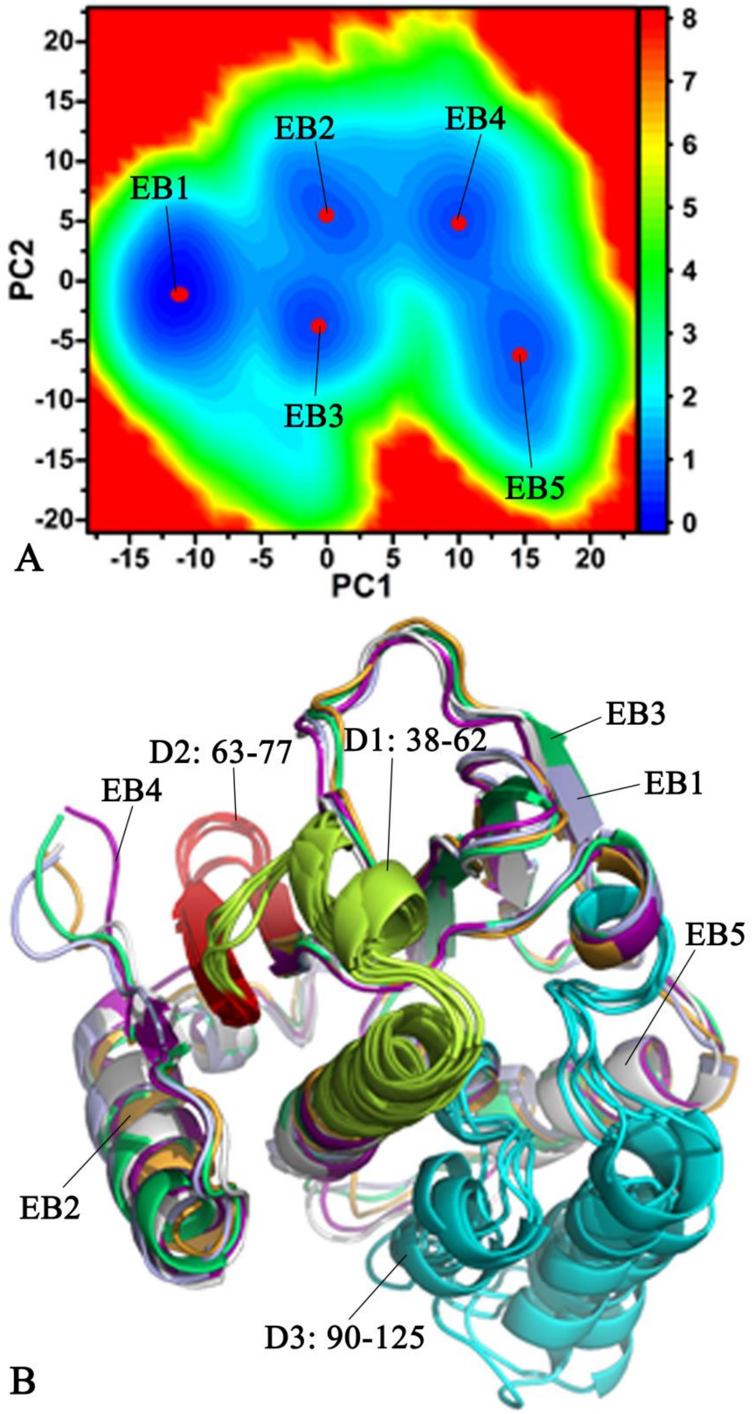

Figure S4. Free energy landscapes and the representative structures of the *apo* HSP90: (A) free energy landscape and (B) structural superimposition of the apo HSP90 trapped at the EB1-EB5.
